# Police Officers’ Attitudes, Intentions, and Stereotyping Towards Mental Health Help Seeking Behaviors

**DOI:** 10.21203/rs.3.rs-6181234/v2

**Published:** 2025-03-20

**Authors:** Suzanne Hakeos, Martina Mueller, Tatiana Davidson, Megan A. Thoen, Michelle Nichols, Sarah Miller

**Affiliations:** Medical University of South Carolina College of Nursing; Medical University of South Carolina; Medical University of South Carolina; Texas Tech University; Medical University of South Carolina; Medical University of South Carolina

**Keywords:** Police officers, Mental health, Help seeking Attitudes, Help seeking Intentions, Mental distress, mental health resources

## Abstract

Police officers have been known to have decreased willingness to seek mental health help. Increasing focus has been placed on enhancing the officer’s mental wellbeing but has this improved the officer’s help-seeking behavior?

**Methods.:**

This cross-sectional quantitative study evaluated officers’ mental health help-seeking attitudes, intentions, and stereotyping of others seeking help using mental distress levels. Data was collected from 337 officers across 22 states.

**Results.:**

Statistical significance was found in 1) age and mental distress, 2) gender on help-seeking attitudes, intentions, and stereotyping, and 3) experience and mental distress. Officers reported more positive help-seeking attitudes, intentions, and stereotyping.

**Discussion.:**

This study supported and contradicted past studies regarding these variables. Officers within this study reported increased officer help-seeking behaviors and less mental distress. Is increased mental health education helping to improve officer help-seeking behaviors, or are officers with lower mental distress willing to participate in mental health research versus those with high mental distress resulting in skewed overall results?

## Introduction

Law enforcement service repeatedly places police officers in high-risk situations that can result in physical and psychological harm.(1–3) Police officers are trained to physically protect themselves and others through self-defense tactics, weapons competencies, and de-escalation tactics.(4–6) Traditionally, officer training focused on prevention of physical versus psychological harm.(7) Over the past decade mental health awareness, education, and services have gained greater importance and recognition throughout the law enforcement population.

In 2014, the President’s Task Force on 21st Century Policing was created to address community and officer trust and safety.(8) Pillar # 6 deals with officer physical and mental wellness and safety based on increased exposure to acts of violence, abuse, accidents, community member and fellow officer suicides, and threats to their own safety while policing.(8–10) The resulting report discusses how four police departments in the U.S. addressed officer safety, and physical and mental wellbeing.(8) Most of the initiatives focused on the officer’s physical health (e.g., heart attack and diabetes prevention, nutritional support, routine exercise), environmental (e.g., exposure, hydration) and vehicular safety (e.g., seat belt use, distracted driving, speed).(8) Unfortunately, the case studies minimally addressed the mental wellbeing of officers, with only two of the four implementing emotional survival, stress management, and mental health help after a traumatic event.(8)

In 2018, the Law Enforcement Mental Health and Wellness Act of 2017 (LEMHWA) was signed into law stressing officer mental wellbeing is just as important as physical health.(11) The U.S. Department of Justice (DOJ) was tasked with compiling the mental health practices and services provided by the U.S. Departments of Defense and Veterans Affairs. Understanding police officers share similar characteristics with military personnel, it was felt effective military services would likely benefit officers. Spence et al.(2019) produced the LEMHWA Report to Congress containing the military’s mental health services and effectiveness of police-centric crisis lines, annual mental health checks, peer mentoring, and confidentiality considerations.(11)

In 2019, Copple et al. produced a report discussing how ten police departments implemented programs to increase the mental wellbeing of their officers, along with the effectiveness of a national call-in hotline.(12) The interventions included mental health screening, resiliency and self-care training, suicide prevention, leadership training, critical incident response teams, peer support, counseling and treatment, therapy animals, and spousal and retiree support. The report was meant to be helpful to other police departments in guiding future mental wellbeing plans and service implementation.

Unfortunately, officer mental wellbeing services in the United States is in its infancy and still severely deficient. Services are not currently regulated or funded, some lack evidence-based research to know if they are effective or not in improving officer mental wellbeing, and are stifled by access barriers.(8,9,11–14) Research has well-established that police officers face significant barriers to help-seeking, including access and use of mental health services. (9,13,14) These barriers include a lack of leadership support, stigma, service and access knowledge deficit, issues with confidentiality, mistrust of mental health professionals, fear of being ridiculed, being viewed as weak, being deemed “unfit for duty,” and ultimately losing one’s career.(9,13,14) Evolving nationwide distrust of law enforcement has added to police officers’ stress by increasing their self-isolation, feelings of unworthiness, and negative self-image, while decreasing help-seeking behaviors.(12,15)

Considering the increasing focus on police officer mental health awareness, societal views on law enforcement, and barriers to officers seeking mental health help, this study aimed at evaluating police officers’ current mental health help seeking attitudes and intentions, and stereotyping of others who seek mental health help using three comparison groups based on the officer’s mental distress level. This cross-sectional quantitative study is part of a larger multi-methods study with an overall goal of understanding officers’ perceptions, knowledge of mental wellbeing services, and willingness to participate in services; qualitative findings will be reported separately.

## Methods

### Participants

IRB approval was obtained through the Medical University of South Carolina. Participants were recruited using snowball sampling through emails which resulted in 337 self-reported sworn, full-time police officers working in 22 of the United States accessing the online REDCap survey.

No compensation was provided for completing the online survey. The initial target survey sample size was 100, which was based on a response rate of 69.4% of police officers completing the online survey and to ensure a sufficient pool of potential interview participants for subsequent qualitative interviews. Following rapid response to the online survey, an IRB amendment was submitted to increase the initial sample size to 875 and to ensure inclusion of all responses with relevant data in the analysis.

Inclusion criteria were self-reported as English speaking, actively working full-time (36 hours a week average) as a sworn police officer, aged 21 years of age or older with at least one year of independent police officer experience in the United States.

Exclusion criteria were currently on temporary disability or suspension.

## Instruments

### Mental Help Seeking Attitudes Scale (MHSAS)

The MHSAS measures participants’ attitudes towards seeking mental health help from a professional mental health provider. The scale consists of nine Likert-type items. The Likert scale ranges from 1–7 (with varying response options between items) and is calculated based on the mean of the nine questions resulting in the highest possible total score of seven. The higher the score the more positive a person’s attitudes are towards seeking help. Cronbach’s alpha was 0.93 (exploratory) and 0.94 (confirmatory) within the U.S. adult population.(16,17)

#### Mental Help Seeking Intention Scale (MHSIS)

The MHSIS measures participants’ intention to seek mental health help from a professional. The scale consists of three Likert-type items. The Likert scale ranges from 1–7 (Extremely likely [7], Moderately likely, Slightly likely, Neutral, Slightly unlikely, Moderately unlikely, Extremely unlikely [1]) and is calculated based on the mean of the nine questions resulting in the highest possible total score of seven. The higher the mean score is towards 7 the greater the intention to seek help for mental health needs. Cronbach’s alpha = 0.94 within the U.S. adult population.(18,19)

##### Help-Seeker Stereotype Scale (HSSS)

(13,20) The HSSS measures participants’ negative stereotyping towards people who seek mental health help from a psychologist. Stereotyping is a set belief that a group of people share the same characteristics, traits, or behaviors ignoring the uniqueness of the individuals within that group. If a person positively stereotypes a person who seeks mental health help, then they view that person and their help seeking behaviors as a good thing (e.g., strong, effective). If they have a negative stereotype of the person and their efforts in seeking help, they will view them poorly (e.g., weak, ineffective). The Likert scale ranges from 1–7 (Strongly agree [7], Agree, Somewhat agree, Neutral agree nor disagree, Somewhat disagree, Disagree, and Strongly disagree [1]) and is calculated based on the mean of the 12 questions. The higher the mean score represents increased negative stereotyping of people seeking mental health help from a psychologist. The closer the mean score is towards 1, the more positive the officer feels towards other people who seek out mental health help from a psychologist. Cronbach’s alpha = 0.93 within the U.S. adult population.(21,22)

To date, this is the only known study to use any of the above three scales in the police officer population.

#### Kessler Distress scale (K-6)

The K-6 measures participants’ psychological distress levels in the general population. The Likert scale ranges from 0–4 for six questions for a sum ranging from 0–24 with up to 11 additional unscored questions depending on participants self-reporting. The original K-6 scale is dichotomized between lower mental distress scored from 0–12 and high mental distress scored from 13–24. The higher the score the more indication there is of serious mental distress. The original K6 was found in the U.S. to have a Receiver Operating Characteristic (ROC)/ Area Under the Curve (AUC) of 0.87–0.88 for Global Assessment of Functioning (GAF) scores = 0–70) and 0.95–0.96 for GAF = 0–50.(23) Initially, the Kessler Distress (K6) scale total score was dichotomized into low (0–12) and high (13–24) mental distress levels for comparison groups, but when the data were reviewed there was an overwhelming percentage of participants (96.8%) that fell within the low distress (0–12) range. There was concern that comparing low to high distress would miss the participants who were higher on the low distress (0–12) scale, overlooking a moderate level distress group. A study by Prochaska et el. (2012) on 50,880 California adult residents found moderate mental distress fell within the greater than or equal to 5 to 12 range using the K-6 scale with an overall ROC = 0.74 and area under each ROC curve (AUC) value of 0.82.(17) Based on these findings for the purposes of this study the Kessler Distress total score was trichotomized using low mental distress (0–4), moderate mental distress (5–12), and high mental distress (13–24).(17)

### Data Collection Procedures

The entire online survey consisted of 5 eligibility questions, 41 questions made up of four scales, and 11 questions assessing demographic characteristics, which were administered using REDCap through the South Carolina Clinical and Translational Science (SCTR) Institute at the Medical University of South Carolina. REDCap and this project was supported, in part, by the National Center for Advancing Translational Sciences of the National Institutes of Health under Grant Number UL1 TR001450. The content is solely the responsibility of the authors and does not necessarily represent the official views of the National Institutes of Health.

### Statistical Analyses

Descriptive analysis of the data was performed in SPSS version 28 to obtain the mean, median, percentile (25th & 75th), percentage, frequency, standard deviation, and standard error for demographic characteristics as appropriate. Chi-square tests evaluated potential associations between categorical variables (department size, gender, marital status, race, military status, ethnicity, and mental distress groups (low, moderate, high). One-way ANOVA or independent t-tests were used to compare continuous variables (age and experience) across mental distress groups (low, moderate, high).

## Results

Of the 337 officers who accessed the REDCap survey, 282 completed the Mental Help Seeking Attitudes Scale (MHSAS) questionnaire (presented first), 262 completed the Mental Help Seeking Intention Scale (MHSIS) questionnaire, 260 completed the Help-Seeker Stereotype Scale (HSSS), 252 completed the Kessler Distress (K-6) questionnaire, and 240 officers completed the demographics section (last section of the survey). Reasons for not completing the entire survey may have included time constraints, confidentiality concerns, mental health stigma, and survey fatigue.

A total of 11 respondents did not provide demographic information (four in the low distress, six in the moderate distress, and one in the high distress groups). One additional respondent did not provide their information regarding gender or department size. For the remaining participants, the mean age was 45 years (range 23–69). Police experience mean was 19.2 years. Males accounted for 78.3% of the population, while females represented 20.4%. Most officers self-identified as White (85.9%), followed by Black officers at 7.1%, 3.3% self-reported as being Hispanic or Latino, and 76.3% reported being married (Table 1).

The mean MHSAS total score was 5.4 (1.1) out of the highest score of 7 correlating with a more positive attitude to seeking mental health help, while the mean MHSIS total score was 4.9 (1.7) out of the highest possible score of 7 correlating with a greater intention to seek mental health help. Participants completing the HSSS had a mean score of 2.2 (1.0) indicating a more positive stereotyping of a person seeking mental health help. This is clinically relevant as officers within this study showed more positive views regarding themselves and others seeking mental health help (Table 2).

The Kessler Distress scale (K-6) mean was 4.8 (3.7) for the 252 participants. The low mental distress group consisted of 141 officers, the moderate mental distress group of 103, and eight participants fell into the high mental distress group. Only 3.2% of the participants reported having high mental distress. Regarding gender and mental distress, females fell almost equally between the low (n = 23) and moderate distress (n = 25) groups, while over half (60.1%) of the males fell within the low distress group (n = 113) (Table 3).

Statistical significance was found in 1) age and mental distress level, 2) gender on mental health help seeking attitudes, intentions, and stereotyping, and 3) years of experience and mental distress levels.

Age was statistically significantly associated with mental distress level both as continuous variable (p = 0.022), and as binary variable dichotomized based on a median age of 46 (< 46 vs ≥ 46; p = 0.025). Results inferred older officers reported lower distress levels by a difference of 1.06. The mean age of the low mental distress group was slightly higher at 46 years of age versus the mean of 43 years of age for both the moderate and high distress groups (Table 3).

Years of police experience and mental distress levels were found to be statistically significant (Table 3). On further review, mean years of police experience were statistically significantly different between the low and moderate distress level groups (p = 0.012), but no significance was found in comparison with the high distress level group (Table 4). Upon further statistical inspection, a linear regression was performed between age, years of experience, and mental distress levels. This showed no statistically significant relationship between the variables of age, experience, and distress level.

Statistically significant associations were observed between gender and mental health seeking attitudes (p = 0.003), intentions (p = 0.050), and stereotyping (p = 0.002). Female officers showed slightly increased mental health help seeking attitudes and intentions, as well as decreased negative stereotyping of others who seek help versus their male counterparts (Table 5).

## Discussion

The purpose was to evaluate police officers’ current attitudes, intentions, and stereotyping of mental health help-seeking behaviors, as well as comparing those concepts between low, moderate, and high mental distress groups. Police officers reported more positive attitudes and intentions towards personal mental health help seeking behaviors, and positive stereotyping of others who seek mental health help.

Officers in this study reported more positive attitudes towards seeking help. Results were opposite from the dissertation findings by Meyer (2000), which found officers’ attitudes to be neutral or indifferent (24), and the study findings by Karaffa and Koch (2016) revealing officers to have more negative attitudes to seeking mental health help. This is likely a result of time since those results were published, as well as increased emphasis on mental health education within the police officer population (12,25,26) and effects of the COVID-19 pandemic on the general population’s mental health needs and awareness.(27)

Although statistically significant in this study, it is unclear whether gender differences are clinically meaningful regarding mental health seeking attitudes, intentions, and stereotyping as no clinically relevant effect size or Minimally Clinically Importance Difference (MCID) has been determined yet for these health domains. Without published standards for these scales, the recommendations of Norman et al. (2003) were used to evaluate MCID based on a half point standard deviation difference. None of the scale results in this study showed the recommended point difference as seen in Table 4, which confirms no clinical relevance between gender and attitudes, intentions, and stereotyping found between the participants in this study.(28)

This study found no statistically significant relationship between gender and mental distress level though the K-6 scores were slightly higher in female officers. Patterson (2003) and Gu et al. (2013) found female officers had higher levels of mental distress over male officers,(29,30) whereas Harnett et al. (2023) found male officers reported higher mental distress over female officers.(31) A study by Haarr and Morash (1999) found female police officers had a 48 percent higher chance of being in the higher occupational stress group,(32) and Kyron et al. (2019) found occupational stress was associated with higher levels of mental distress.(33) This study did not measure occupational stressors, though it would be interesting to know if the level of occupational stress an officer reports correlates to their gender, and help-seeking attitudes, intentions, and stereotyping.

Officers in our study showed more positive intentions towards seeking help for personal mental health concerns. This supports a portion of the findings by Karaffa and Tochkov (2013) in which officers reported feeling they are personally more willing to seek mental health treatment versus their peers.(34) This could be a result of increased officer mental health education and awareness,(35–37), possible change in the traditional police culture (38) with newer generations of officers joining the force, leadership support,(13,37) and implementation of departmental mental health resources.(39)

Officers reported more positive stereotyping of others who seek mental health help. This is supported by Hanafi et al. (2006) in which officers reported reduced stereotyping/stigmatization of community members with mental health issues following a Crisis Intervention Team education training.(40) With the increased mental health emphasis both for officers and community members over the last decade, it is likely that improved educational exposure and awareness through the media and within departments has helped to decrease negative stereotyping of help seekers.

A statistically significant relationship was also found regarding age and mental distress levels, showing the older the officer the less mental distress they reported. In support of this, Patterson (2003) found increased age of the officer was associated with increased police experience, rank, and decreased “stressful work events” resulting in less mental distress.(29) This is contrasted by Arial et al. (2009) who found older officers had more psychiatric symptoms than younger officers.(41) Based on the skewed distribution of participants in the low distress (n = 141) and moderate distress groups (n = 103) compared to the high distress group (n = 8), no clinical relevance was placed on the 3-year mean age difference using group comparison. On further inspection, there was no statistically significant relationship between police officers age, years of experience, and mental distress score which supports age and distress not being clinically relevant in this sample.

There was a statistically significant association between years of police experience and mental distress groups, but with further evaluation there was no clinically relevant pattern seen between a mean of 21 years of experience in the low distress group, 17 years in the moderate distress group, and the high distress group mean at 19 years of experience. The skewness in the numbers between the mental distress groups made it difficult to say true statistical significance was found, since 234 participants fell within the low to moderate mental distress groups and only seven in the high mental distress group.

On another note, officers can be suspicious or distrustful of outsiders,(34) which can result in decreased participation with mental health professionals and researchers.(14) Known fears (e.g., confidentiality concerns, being viewed as weak, loss of job) may deter officers with high mental distress from participating in surveys or answering questions about personal mental health. This study’s principal investigator is not a police officer, but her spouse of 18 years is, which may have provided an increased level of trust, access, recruitment, and survey completion support offered by officers and departments.

It is also likely officers participating in mental health research have more positive personal views of help seeking, increased leadership support, and lower levels of stigma. This could be related to increased governmental focus on mental health education and service access to officers. Currently, resources are not available in all U.S. police departments. The availability of mental health resources within departments is dependent on several factors such as leadership support, stigma, resource availability, financial resources, department size and type (urban vs rural, federal vs city).(13)

## Conclusion

This is the first study to evaluate the combination of officers’ attitudes, intentions, and stereotyping regarding mental health help seeking behaviors using mental distress levels and use of the MHSAS, MHSIS, HSSS within the police officer population. Overall, the study results provide positive insight into officers’ current attitude and willingness to seek mental health help and support others who seek help. It is likely, based on these results, that officers would be more inclined to: 1) seek help and participate in mental health services if barriers are removed, and 2) provide encouragement and support to fellow officers to seek help.

### Future Recommendations

Should include: 1) continued research into police-centric mental wellbeing services to produce evidence-based guidelines for police departments to implement proven mental health services to increase the mental wellbeing of officers, 2) funding resources for service implementation based on department financial need (e.g., small departments versus large departments), 3) an MCID regarding the Mental Health Seeking Attitudes, Mental Help Seeking Intentions, and Help-Seeker Stereotype scales, 4) barrier reduction techniques (e.g., increased leadership support, stigma reduction, normalizing mental wellbeing services) to improve officer willingness and access to mental health services without fear, and 5) further research into if gender plays a part in officers’ attitudes, intentions, and stereotyping of help seeking behaviors.

### Strengths and Limitations

Strengths included 1) a large sample of full-time, sworn police officers from 22 States within the U.S., and 2) Demographics provided matched closely to the national average within the law enforcement population.

Limitations included 1) not all participants completed all the scales or demographic questions which could have been related to survey fatigue, time constraints, interruptions, fear related to discussing mental health or confidentiality concerns and resulted in missing data. There was a steady decrease in completion rates of the scales starting from the first scale through the demographics section provided at the end of the survey, 2) skewness of mental distress groups with only 7 participants in the high distress group, and 3) type of police department was accidentally omitted on the survey.
